# Polypoidal Choroidal Vasculopathy in Congolese Patients

**DOI:** 10.1155/2020/4103871

**Published:** 2020-01-18

**Authors:** Nelly N. Kabedi, David L. Kayembe, Gloria M. Elongo, Jean-Claude Mwanza

**Affiliations:** ^1^Department of Ophthalmology, School of Medicine, University of Kinshasa, Kinshasa, Democratic Republic of the Congo; ^2^Department of Ophthalmology, School of Medicine, University of North Carolina at Chapel Hill, Chapel Hill, North Carolina, USA

## Abstract

**Purpose:**

Polypoidal choroidal vasculopathy (PCV) is a visually debilitating disease that mostly affects people of African and Asian heritage. Indocyanine green angiography (ICGA) is the recommended exploratory method for definitive diagnosis. The disease has been extensively described in Asians and Caucasians, but not in Africans. This study was conducted to document the clinical presentation and optical coherence tomography features of polypoidal choroidal vasculopathy (PCV) in Congolese patients.

**Methods:**

A prospective case series of patients with PCV was performed between January 2017 and June 2019. Routine ocular examination was performed including best corrected visual acuity measurement, slit-lamp examination, dilated direct fundoscopy, and spectral domain optical coherence tomography (OCT). The diagnosis was based on a combination of clinical and OCT signs.

**Results:**

Fourteen patients were diagnosed with PCV during this period. The average age was 64.7 ± 6.9 years. There were 8 females. Ten (71.4%) patients had systemic hypertension. Most patients (*n* = 9, 64.3%) had bilateral involvement. Blurred vision was the most common complaint (71.4%). The main clinical presentation was subretinal exudates, seen in 19 (82.6%) eyes of 11 (78.6%) patients and subretinal hemorrhage in 10 (43.5%) eyes. Macular localization was found in 16 eyes (69.5%) of 12 (85.7%) patients. Drusen were observed in 35.7% of the patients. On OCT imaging, thumb-like pigment epithelial detachment and subretinal exudation were the most frequent features, observed in 92.9% and 71.4% of the patients, respectively.

**Conclusions:**

PCV in Congolese patients showed features that are more similar to those observed in Caucasians. In this setting where indocyanine green angiography is not available, OCT facilitates the diagnosis of PCV.

## 1. Introduction

Polypoidal choroidal vasculopathy (PCV) is an abnormal proliferation of choroidal vessels that may lead to recurrent serous and hemorrhagic exudation [1]. Since the first report on the disease by Yannuzzi et al. [2], several refinements have been made to its description over the years. Although indocyanine green angiography (ICGA) remains the gold standard imaging modality for its diagnosis [3], significant advances in imaging techniques have contributed to enhance our knowledge of the disease. In particular, the advent of optical coherence tomography (OCT) in recent years has allowed noninvasive quantitative assessment and longitudinal monitoring of the related structural changes that cannot be observed on ICGA. Observation of these changes has led to new diagnostic criteria based on OCT features [4–6]. The rapid widespread use of OCT has notably facilitated the diagnosis of the disease in settings where ICGA is not available [4, 7]. The treatment of PCV has been the subject of several studies in the past, with a general consensus that combining photodynamic therapy (PDT) with anti-VEGF intravitreal injections is the best therapeutic option [1, 8, 9].

Although PVC has been reported to be more frequent in people of African heritage, most available data have come from studies conducted in Europe and the USA [10–12]. While it is assumed that blacks in sub-Saharan Africa (SSA) are prone to developing PCV, data to support such an assumption are largely lacking. Indeed, no such information exists for the Democratic Republic of Congo (DRC), and an extensive search on various library databases retrieved only one published study from Nigeria [13]. In that report, the authors described the clinical features, treatment, and functional outcome in 10 patients seen over a 6-year period. The diagnosis was based on clinical fundus examination without assistance of either ICGA or OCT.

Because SSA is very diverse genetically and environmentally with the resulting wide epigenetic variation, one should not necessarily expect epidemiological and clinical features to be similar across SSA regions, especially for diseases where epigenetic mechanisms are believed to play a pathogenic role [14]. For this reason, country-specific data are important. Herein, we report the clinical features of PCV in 14 Congolese patients seen at the University Hospital of Kinshasa (UHK), DRC.

## 2. Subjects and Methods

A prospective case series of 23 eyes of 14 patients diagnosed with PCV was conducted in the Department of Ophthalmology, UHK, from January 2017 to June 2019. This study was approved by the Institutional Review Board of the School of Public Health, School of Medicine, University of Kinshasa, and adhered to the tenets of the Declaration of Helsinki. We excluded patients who fulfilled one or more of the following criteria: (1) evidence or a history of other causes of choroidal neovascularization (i.e., age-related macular degeneration (AMD), degenerative myopia, central serous chorioretinopathy, and angioid streaks), (2) proliferative diabetic retinopathy, (3) a history of chorioretinitis, (4) extensive retinal scars and/or fibrosis, (5) previous retinal surgery, and (6) media opacities preventing good visualization of retinal lesions. Patients were all submitted to an interview to collect information about the history of the disease (chief complaint, duration of the complaint, any treatment received prior to presentation to our visit, and time interval of onset between eyes in case of bilateral involvement) and current and past systemic conditions. They also underwent body mass index (BMI) determination, resting blood pressure measurement, an ophthalmic examination and spectral domain OCT imaging in both eyes, and laboratory testing. ICGA was not available.

The eye examination included decimal best corrected visual acuity (BCVA) measurement on a Monoyer chart, slit-lamp examination, Goldmann applanation intraocular pressure (IOP) measurement, and dilated direct ophthalmoscopy. Ophthalmoscopic signs of PCV included subretinal reddish-orange nodules, retinal exudates, subretinal hemorrhage, serous neurosensory detachment, and/or serous or hemorrhagic pigment epithelial detachment (PED). These lesions were described with regard to their location (peripapillary, macular, interpapillary-macular, and peripheral).

Both eyes of each patient were also imaged through dilated pupils with a 3D-OCT 1000 (Topcon Corp., Tokyo, Japan) using the macular 512 × 128 Cube (6 mm × 6 mm) scanning mode. Only good quality scans, defined as those with a signal strength equals to or greater than 45 per manufacturer recommendation and without artifacts (i.e., blinking, motion, out-of-range, mirror) and segmentation errors, were kept and read. All OCT scans were evaluated by the same ophthalmologist (N.N.K.) with input from J.C.M and reread remotely by an external fellowship-trained retina specialist (Jessica G. Shantha, Emory Eye Center, Emory University, Atlanta, USA) for the following signs: (1) one or multiple thumb-like, sharp-peaked, M-shaped or notched PED, (2) double layer sign (DLS), (3) subretinal fluid (SRF), and (4) hyporeflective luminen with hyperreflective contour [4, 5]. The presence of at least 2 clinical signs and 3 OCT signs was suggestive of PCV.

The panel of laboratory testing consisted of hemoglobin (Hb), white blood cell (WBC) count and differential, erythrocyte sedimentation rate (ESR), C-reactive protein (CRP), fasting serum glucose (FSG), total lipids, triglycerides, total cholesterol, low-density lipoproteins (LDL), and high-density lipoproteins (HDL).

## 3. Results

This series included 6 men and 8 women. The mean age was 64.7 ± 6.9 years (range 51 to 75 years). The mean duration of symptoms was 3.3 ± 3.4 years (range 3 months to 12 years). At the time of presentation at our clinic, all patients had been seen previously by other providers, 4 (28.6%) of them were diagnosed as having age-related macular degeneration (AMD), whereas the remainder had no clear diagnosis. Systemic hypertension was present in 10 (71.4%) patients, 2 patients had diabetes, 1 patient had herniated disc, and another one had rheumatoid arthritis. Of the 23 eyes, 2 (1 patient, 8.7%) were glaucomatous, 4 (2 patients, 17.4%) had epiretinal membranes, 2 (1 patient, 8.7%) had nonproliferative diabetic retinopathy, and 2 (2 patients, 8.7%) had undergone cataract surgery. None of the patients had a history of smoking habit. Eight (57.1%) of the patients were either overweight or obese based on BMI.

Table 1 lists the demographic, clinical, and OCT findings. Patients formulated the main reason for presentation at the clinic as blurred vision (*n* = 10), vision loss (*n* = 4), scotoma (*n* = 1), and metamorphopsia (*n* = 1). Bilateral involvement was seen in 9 (64.3%) patients. Of the 23 diseased eyes, 16 (69.6%) had BCVA limited to count fingers or lower; the remainder of the eyes had a BCVA of 0.1 or greater. On fundus examination, exudates were observed in 11 patients (78.6%) and subretinal hemorrhage (SRH) in 10 patients (71.4%). Five (35.7%) patients had drusen and 2 patients had reddish-orange lesions. Two (14.3%) patients had hemorrhagic PED. The lesions were localized in the macular area in 12 (85.7%) patients, among whom 5 (35.7%) patients had lesions extending to the peripapillary area as well. The lesions were solely peripheral in 1 (7.1%) patient. The mixed variety of PCV (hemorrhagic and exudative) was seen in 9 patients (64.3%), the exudative only in 3 patients (21.4%), and the hemorrhagic only in 2 patients (14.3%).

**Figure 1:**
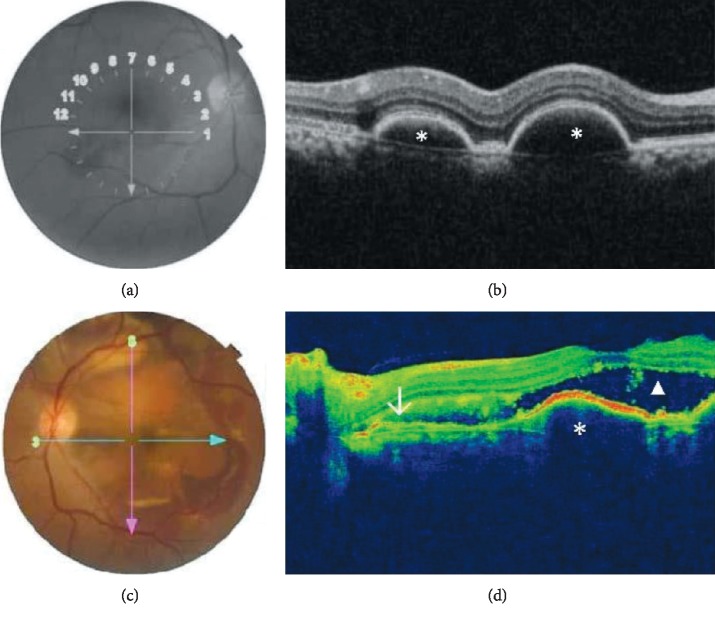
Fundus photos and OCT scans for a patient with bilateral PCV. (a) Fundus photo of the right eye which displays temporal subretinal hemorrhage with central subretinal fluid. (b) OCT shows multiple pigment epithelial detachments (asterix). (c) Fundus photo of the left eye displays a red-orange nodule in the superior macula and multiple areas of subretinal hemorrhage within the temporal macula and extending to the arcades. (d) Corresponding OCT image shows a temporal branching vascular network (arrow), with subretinal hyperreflectivity, serosanguineous pigment epithelial detachment (asterix), and subretinal fluid (triangle).

**Figure 2:**
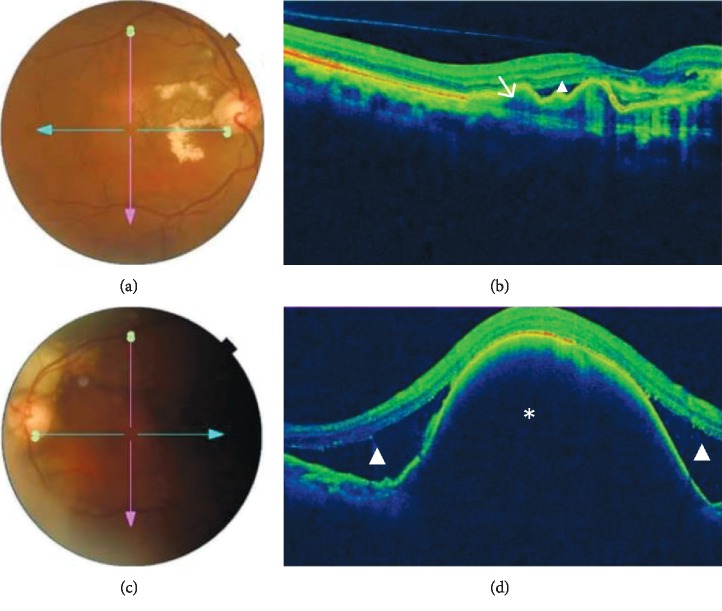
Imaging of a patient with bilateral PCV. (a) Fundus photo of the right eye shows red-orange nodules within the superior macula with hard exudates and resolving subretinal hemorrhage. (b) Corresponding OCT shows subretinal fluid with branching vascular network (arrow) with classic double layer sign. (c) Fundus photo of the left eye shows subretinal hemorrhage with resolving hemorrhage superior to the optic nerve. (d) OCT displays a large pigment epithelial detachment (asterix) which is characteristic finding in patients with PCV.

**Figure 3:**
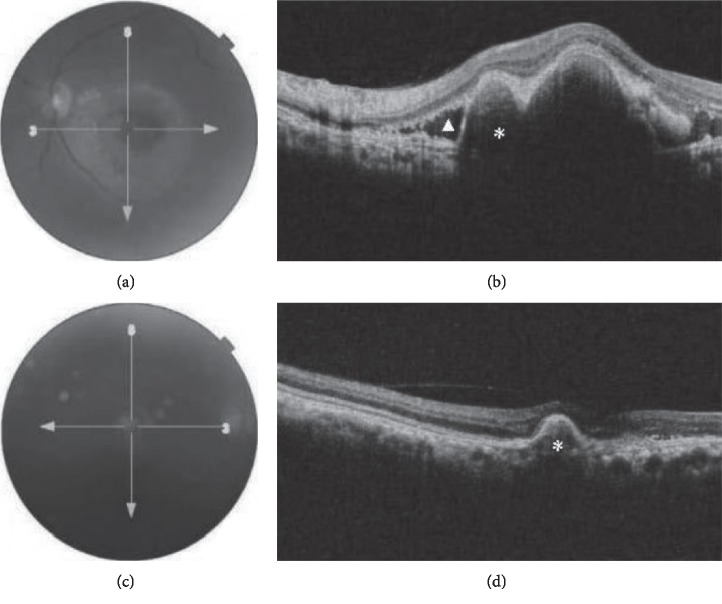
(a) Fundus photo of the left eye in a patient with unilateral PCV displaying exudates, subretinal hemorrhage, and a pigment epithelial detachment. (b) OCT shows subretinal fluid with subretinal hyperreflectivity (triangle), a pigment epithelial detachment (asterix) with a thumb-like projection and notch which is characteristic of a polyp lesion. (c) Fundus photo of the right eye. (d) OCT shows pigment epithelial detachment (asterix) and adjacent retinal atrophy.

**Figure 4:**
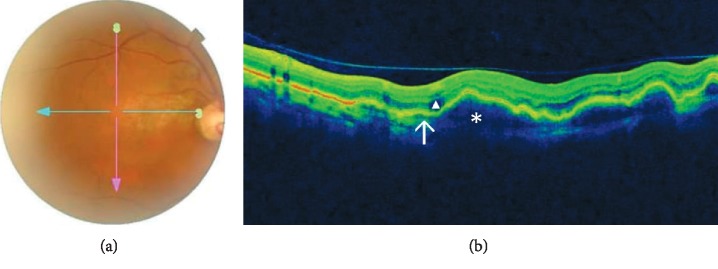
(a) Fundus photo of the right eye in another patient with unilateral PCV showing red-orange nodules and in the posterior pole and subretinal fluid and a pigment epithelial detachment. (b) OCT shows few subretinal fluid (triangle), multiple pigment epithelial detachments (asterix) and notched thumb-like elevation, and branch vascular network (arrow).

OCT demonstrated thumb-like PED in 13 (92.9%) patients and subretinal exudation in 10 (71.4%) patients. Polypoidal lesions were observed in 92.9% of the patients, liquid pockets and DLS were present in 5 (35.7%) patients each, and sharp-peaked PED and multiple PED in 4 (28.6%) patients each. A notch sign and hard exudates were visualized on scans of 3 (21.4%) patients each. PED break was seen in one patient. Figures 1–4 show OCT fundus photos and scans of 4 selected PCV patients. A branching vascular network (BVN), represented by the DLS, was visible in 5 (35.7%) patients.

The serum biochemical profile of the patients showed elevated CRP in 3 (21.4%) patients, elevated urea in 2 (14.3%) patients, and within normal levels of creatinine in all patients. Fasting glucose level was elevated in 2 (14.3%) patients. One (7.1%) patient had elevated levels of total proteins. Total lipids were diminished in one (7.1%) but elevated in 2 patients (14.3%). Triglyceride levels were elevated in 2 (14.3%) patients. One patient had higher than normal levels of total cholesterol, all other had normal levels. HDL-cholesterol was lower than normal in 2 (14.3%) patients, whereas serum LDL-cholesterol was elevated in 3 (21.4%) different patients.

## 4. Discussion

The mainstay of PCV diagnosis is ICGA [11, 15]. However, ICGA is invasive, its use has been restricted due to allergy [15], and it is not available in most countries in SSA. In addition, ICGA is time-consuming (i.e., 30 minutes), and additional resources are required for its completion compared to OCT. Moreover, two recent studies [4, 5] found OCT-based methods to diagnose PCV highly sensitive and specific to differentiate PCV and other causes of choroidal neovascularization. Because ICGA is not always available, Shantha and Kokame [6] pointed out recently that consideration should be given to other imaging modalities (i.e., OCT, en face OCT, and OCT angiography) in settings where IGGA is not feasible. It is for the above reasons that we investigated the clinical and OCT features of Congolese PCV patients. This is, as far as we know, the first characterization of PCV clinically and OCT-wise in SSA.

Prior to being seen by us, all patients in this study had visited other providers, but none had been diagnosed as having PCV. Instead, 4 of them were misdiagnosed as having AMD. This observation confirms that PCV is underdiagnosed and often misdiagnosed as AMD. While PCV may be well known elsewhere, misdiagnosis in our setting results from the lack of awareness of the disease and lack of adequate diagnostic tools. Misdiagnosing PCV as AMD is not uncommon as evidenced by available data. In Italian patients, Scassellati-Sforzolini et al. [16] found that 9.8% of those diagnosed with presumed exudative AMD had PCV. Such a prevalence was 8.2% among Greek [17] and 10.6% among Brazilian patients [18]. In Danish patients, Ilginis et al. [19] reported that 8% of those initially diagnosed with neovascular AMD on fluorescence angiography ultimately had PCV after ICGA was performed. A recent meta-analysis that included data from 11 studies estimated the prevalence of PCV to be 8.7% in European white populations with exudative AMD. In Asian populations, the prevalence rates of 22.3%, 23%, 24.6%, and 30.8% were reported in China [20], Japan [21], Korea [22], and Thailand [23], respectively. It will be interesting to document such rates in SSA where both the incidence and prevalence of presumed AMD are on the rise.

The mean age in this series was 64.7 years, which is consistent with previous reports that PCV is mostly seen during the 7^th^ decade of life. Although PCV has been described in patients in their 50s [13, 24], the mean age of European PCV patients varied between 65.4 and 77.3 years in studies conducted in Belgium [25], Denmark [19], Germany [26], Greece [17], Italy [16], and Switzerland [27]. Across Asian countries, PCV has been reported to affect patients with a mean age range of 65.1–74.5 years in China [20, 28–30], 63–67.8 years in Korea [22, 31, 32], and 65.7–71.9 years in Japan [21, 33–35]. The multiracial cohort in Alasil et al.'s study [10] had a mean age of 69.4 years and, interestingly, no statistically significant difference was observed between blacks (71.1 years), whites (67.8 years), and Asians (67.8 years). Upon close review of the above studies, it appears PCV often occurs in the 7^th^ and 8^th^ decades of life.

The question whether PCV affects predominantly one sex over the other has generated varying findings across studies. We found a female predominance (57.1%) in the present series. The predominance of female in black PCV patients was also reported among Nigerians (80%) [13], Afro-Caribbean from the French West Indies (71.4%) [36], and in a combined sample of African-Americans and blacks from the United Kingdom (UK) in Alasil et al.'s study (67%) [10]. Similarly, studies in Caucasians have consistently found females to be more affected than males [10, 16, 17, 19, 25–27]. On the contrary, a review of studies in Asian and Middle East populations shows that men are commonly more affected than women [20, 24, 28, 29]. Thus, there seems to exist a clear contrast in the way PCV affects men and women in Asia vs. outside Asia. Surprisingly, associative studies failed to find a significant association between sex and PCV [1, 30, 37–39].

Bilateral cases (64.2%) with predominantly macular localization (85.7%) outnumbered unilateral ones in the present study, in line with study findings in black populations in Nigeria [13] and the French West Indies [36] where all cases were bilateral. Other available data show heterogeneity in laterality and location of the involvement. In Asian cohorts, most cases are unilateral (75.9%–97.4%) with a predominantly macular location [21, 22, 24, 29, 30, 35, 40], similar to the pattern observed in Italian [16] and Brazilian [18] cohorts. A study performed jointly in the USA and the UK that included blacks, Caucasians, and Asians also revealed that PCV was mostly unilateral (79% in blacks, 81% in Caucasians, and 79% in Asians) and more frequently located in the macular region (69.4% in blacks, 84.2% in Caucasians, and 78.3% in Asians) [10]. While the disease was more frequently bilateral in Belgian (61%) and Greek patients (54.5%), the highest proportion in the former had macular (48.9%), whereas the latter mostly had peripapillary (68.2%) disease. It is not clear why the disease is mostly unilateral in African-Americans and African-English but bilateral in Africans. A plausible explanation is that it might result from the genetic difference between these two groups. While Africans in SSA have remained genetically pure, those from slavery are genetically different as a result of interracial mixing and the influence of epigenetics.

Various systemic risk factors have been associated with PCV to varying degrees [1, 9, 41]. Among them, systemic arterial hypertension (HTN) has been singled out in some reports with proportions of patients with HTN ranging between 20% and 70% [30, 36–39, 42–47]. We report herein a rate of 71.4%, which is on the higher end of what has been reported. The difference between our finding and those of past studies as well as across previous studies likely reflects differences in methods for collecting information on HTN. While we measured blood pressure in our patients, others have relied on patients' accounts, which can sometimes be unreliable. Our high rate will certainly need corroboration by future studies in this same setting, in other SSA countries, and in multiracial populations outside Africa. It also may actually be a snapshot of the HTN trend in the general population. Indeed, blacks are known to be at greater risk for elevated blood pressure with earlier onset than Caucasians [48, 49]. A specific reason to our population may be the high prevalence of HTN and uncontrolled HTN in Kinshasa [50]. However, it is worth noting that two studies in Singapore, a large hospital-based case-control study [37] and a population-based case-control study among participants of the Singapore Epidemiology of Eye Diseases Program and the Asian AMD Phenotyping Study [51], the Beijing Eye Study [30], and two other studies in Japan [38, 52] failed to find a significant association between HTN and PCV, thereby contributing to the ongoing debate on the putative role of HTN in PCV development. On the contrary, Kikuchi et al. [45] reported a significantly higher proportion of people with HTN among PCV patients than among controls. Among other potential risk factors, no patient had a history of cigarette smoking in our cohort. The association of cigarette smoking habit with PCV has yielded conflicting findings. In Japan, this association was significantly positive in the Hisayama Study [52], but nonexistent in two other cohorts [38, 39]. The Beijing Study [30] also reported a lack of such an association, contrasting with findings of a Singaporean study where smoking conferred a 4.2-fold greater risk for PVC [37]. In the Singapore Epidemiology of Eye Diseases Program [51], subjects with higher BMI were more likely to have PCV, contrasting with findings of two Japanese studies [45, 52]. Because of the design of the present study, we cannot say with certainty whether higher BMI (57.1%) is a risk factor for PCV. Diabetes has also been investigated for its association with PCV. It is unlikely that such an association existed in our cohort, where only one patient was diabetic. A review of existing data shows that there is yet to be a single study revealing a positive association between diabetes and PCV [21, 30, 37–39, 45, 52, 53]. Only 21.4% of the patients in the present study had elevated CRP. Higher CRP levels have been significantly associated with PCV in one case-control study [45], but the association was not observed with either PCV or AMD in another investigation [51]. Serum total cholesterol was within normal range in all 14 patients, which may be consistent with the lack of its association with PCV reported by Fujiwara et al. [52] and Li et al. [54]. These studies also reported no association between PCV and either serum LDL- or HDL-cholesterol. While 2 of our patients had lower serum levels of HDL-cholesterol, studies on the association of PCV with HDL-cholesterol levels have produced inconsistent findings. Fujiwara et al. [52] as well as Li et al. [54] failed to find an association, whereas Cheung et al. [51] and Fan et al. [55] reported that higher levels of HDL-cholesterol were significant risk factors for PCV.

PCV classically presents on fundus examination as an orange-red nodule in the macula or peripapillary region, associated with serosanguineous PED without drusen [1, 12]. Compared to our cohort where most patients had the mixed variant, others found either the hemorrhagic [13, 29, 36, 56] or the exudative [8, 21, 57] type to be more frequent. This suggests a variable pattern with regards to what the predominant form of PCV is. The proportion of our patients with exudates (78.5%) is higher than reported by Chang et al. (62.6%) [4], Kwok et al. (59.1%) [29], and Yeung and Chen (62.8%) [57]. Because subretinal exudate is a sign of chronicity, our results suggest that the disease has been present for a long period of time prior to presentation at our clinic, likely in a quiescent state. Indeed, a quiescent PCV has been described where exudation and bleeding are absent, with preservation of vision until events such as bleeding and PED occur [1, 9]. Thus, the delayed diagnosis of the disease, which prevented early adequate management, enabled the progression of the disease towards chronicity.

Although drusen are not part of the classical definition of PCV, it has now been recognized that it is not unusual to find them in conjunction with PCV lesions. They were observed in the macular area in 35.7% of the patients in the present series. In other studies among blacks, none of the 10 patients in Nigeria [13] and 50% of the 26 eyes in the French West Indies cohort had drusen [36]. It is important to note that the diagnosis of PCV in the Nigerian cohort excluded the presence of drusen, which explains why no drusen were found. In Caucasians, no drusen were found in the UK and rates of 14.7% and 33.3% were reported in France [58] and Belgium [25], respectively. Contrary to the long-held belief that drusen were not present in Asians, a review of published data indicates that drusen were observed in 9.1%–54.5% in Chinese [28, 29, 56], 23% in Japanese [59], 2%–12% in Koreans [32, 60, 61], and 8.1% in Indians [62]. Evidence also suggests that unaffected fellow eyes of PCV patients show drusen in proportions ranging between 2% and 35.8% [12, 16, 17, 20, 25, 56, 60, 62]. Thus, PCV in black Africans may present with or without drusen.

Based on fundus examination and photography, the diagnosis of PCV could only be suspected in 12 patients and was probable in 2 more patients. The addition of OCT helped uncover typical PCV features and was therefore critical in establishing the diagnosis, in particular by showing the thumb-like elevation of the RPE, which represents polypoidal lesion, and DLS which corresponds to the VBN. Thus, although ICGA is the recommended diagnostic test for the diagnosis of PVC [3], conventional OCT is a reliable alternative, particularly where ICGA is not available or feasible. As OCT is slowly becoming increasingly available in SSA, it is expected that more cases of PCV will be diagnosed. However, this will also require local ophthalmologist to be aware of the disease. More importantly, emphasis should be given to en face OCT, which is available on most OCT devices after reconstructing the B-scans. OCT en face images help visualize the PCV lesions in retinal layers and thus provide an idea on the extent of the disease. Kokame et al. [63] compared en face OCT and ICGA images in a series of eyes with PCV. They reported that the former was as effective as the latter in helping visualize PCV lesions while also providing a better analysis of the extent of the lesions. They concluded that en face OCT was an effective alternative in the absence of ICGA. Other studies have also suggested that en face OCT is a good diagnostic method to visualize PCV-related morphological changes [64–66].

## 5. Conclusions

In conclusion, we have provided the initial clinical and OCT characterization of PCV in Congolese patients. In this population, PCV seems to affect more females than males in their mid-60s and is mostly bilateral with lesions predominantly located in the macula. PED with subretinal serohemorrhagic collection is the most frequent phenotype form, and drusen may also be seen. These attributes resemble more the features described in Caucasians than Asians. This study reaffirms that in the absence of ICGA, conventional OCT is a reliable tool for establishing PCV diagnosis, particularly in patients presenting subretinal hemorrhage and/or exudation of unknown origin.

## Figures and Tables

**Table 1:** Demographics and clinical and OCT findings.

Patient	Age	Sex	Main complaint	Symptom duration	VA at presentation (OD/OS)	Eye	Fundus examination	OCT findings	Localization
1	63	F	Visual loss	4 years	1.0/HM	OS	SRH, HPED	Thumb-like PED, sharp-peaked PED, notch PED, polyp lumen, SRE	Peripapillary
2	60	M	Blurred vision	3 months	0.2/0.4	OU	Reddish orange lesion, exudates	DLS, thumb-like PED, SRE, polyp lumen	Peripapillary, macular
3	67	F	Visual loss	3 years	CF/0.2	OU	Exudates, SRH	DLS, thumb-like PED, fibrosis, SRE, liquid pockets	Macular
4	75	M	Visual loss	8 years	CF/CF	OU	Exudates, SRH	Thumb-like PED, SRH, hard exudates, SRE	Macular
5	63	F	Blurred vision	9 months	1.0/CF	OU	Drusen, exudate, SRH	Multiple thumb-like PED, sharp-peaked PED, SRE, DLS, polyp lumen	Macular
6	73	F	Blurred vision	12 years	CF/CF	OU	Exudates, SRH	Large PED, liquid pockets, SRF, DLS	Macular
7	59	M	Blurred vision	5 years	0.2/NLP	OD	Exudates	Thumb-like and sharp-peaked PED, hard exudates, liquid pockets, SRE, polyp lumen	Peripheral
8	69	F	Visual loss	8 months	CF/0.5	OU	Drusen, exudates	Thumb-like and sharp-peaked PED, hard exudates, liquid pockets, SRE, polyp lumen	Macular, peripapillary
9	51	F	Blurred vision	3 months	1.0/HM	OS	Drusen, SRH	Multiple PED, thumb-like PED, notch sign, SRE, RPE break	Macular
10	64	M	Blurred vision	2 years	1.0/CF	OU	Reddish orange lesion, drusen, SRH, exudates, HPED	Multiple PED, sharp-peaked PED, large thumb-like PED, SRE, notch sign	Peripapillary, macular
11	74	M	Blurred vision	3 years	CF/0.7	OD	Exudate	Thumb-like PED, SRE, polyp lumen	Macular
12	70	M	Blurred vision	1 year	HM/CF	OU	SRF, SRH	Thumb-like PED, liquid pockets	Peripapillary, macular
13	68	F	Blurred vision	1 year	0.8/HM	OS	Exudates, SRH, drusen	Multiple thumb-like PED, liquid pockets, DLS	Peripapillary, macular
14	61	F	Blurred vision	6 years	CF/0.1	OU	Exudates, SRF, SRH	Thumb-like PED, liquid pockets, polyp lumen	Peripapillary, macular

F: female; M: male; VA: visual acuity; OD: right eye; HM: hand motion; CF: count fingers; NLP: no light perception; OS: left eye; OU: both eyes; SRH: subretinal hemorrhage; PED: pigment epithelial detachment; SRF: subretinal fibrosis; SRE: subretinal exudation; DLS: double layer sign; HPED: hemorrhagic pigment epithelial detachment.

## Data Availability

The dataset of this study may be available from the corresponding author upon reasonable request.
